# Inhibitory effect of *Lacticaseibacillus paracasei* VCare-360 on *Candida albicans* in mice with vaginal candidiasis

**DOI:** 10.3389/fcimb.2026.1829740

**Published:** 2026-09-24

**Authors:** Duo Han, Xin Yang, Xiao Hui Zhao, Yu Xing Zheng, Yue Jian Mao, Xiao Wei Zheng, Juan Yang, Jia Lun Chen

**Affiliations:** 1 Global R&D Innovation Center, Inner Mongolia Mengniu Dairy (Group) Co.Ltd., Hohhot, China; 2 Microbial Innovation and Research Center, Nutrition & Health Research Institute, COFCO Corporation, Beijing, China

**Keywords:** *Candida albicans*, antifungal activity, *Lacticaseibacillus paracasei*, probiotic, vaginal candidiasis

## Abstract

**Background:**

Vaginal candidiasis (VC), mainly triggered by *Candida* species, ranks among the most‑common reproductive‑tract infections worldwide. Over‑utilization of antibiotics and antifungal medicines aggravates fungal drug‑resistance, which makes it imperative to explore novel alternative therapies. This work intended to investigate the anti‑*Candida‑albicans* activity of *Lacticaseibacillus paracasei* VCare‑360.

**Methods:**

The in‑vitro capacities of the strain to produce hydrogen peroxide and lactic acid and its antifungal activity were tested. A vaginal‑epithelial‑cell model was adopted to evaluate bacterial competitive adhesion and the regulation of pro‑inflammatory cytokine secretion. A mouse VC animal‑model was constructed to assess vaginal mucosal repair, inflammatory‑factor levels, myeloperoxidase activity, as well as vaginal and faecal microbiota composition after probiotic intervention.

**Results:**

*L. paracasei* VCare‑360 competed against *C. albicans* for vaginal‑epithelial‑cell adhesion and suppressed fungal colonization. It lowered the release of pro‑inflammatory cytokines *in-vitro*. In mice, this probiotic alleviated vaginal epithelial exfoliation, repaired damaged vaginal mucosa and relieved local inflammation (*p* < 0.05). It also decreased pro‑inflammatory cytokine concentrations and myeloperoxidase activity in blood and vaginal tissue. Sequencing results demonstrated that VCare‑360 enriched the relative abundance of *Lactobacillus* and cut down vaginal *Escherichia-Shigella*.

**Conclusion:**

*Lacticaseibacillus paracasei* VCare‑360 can inhibit *C. albicans* infection, modulate vaginal flora and restore the vaginal mucosal barrier, and serves as a promising probiotic candidate for the treatment of *Candida*‑induced vaginal candidiasis.

## Graphical Abstract

**Figure:**
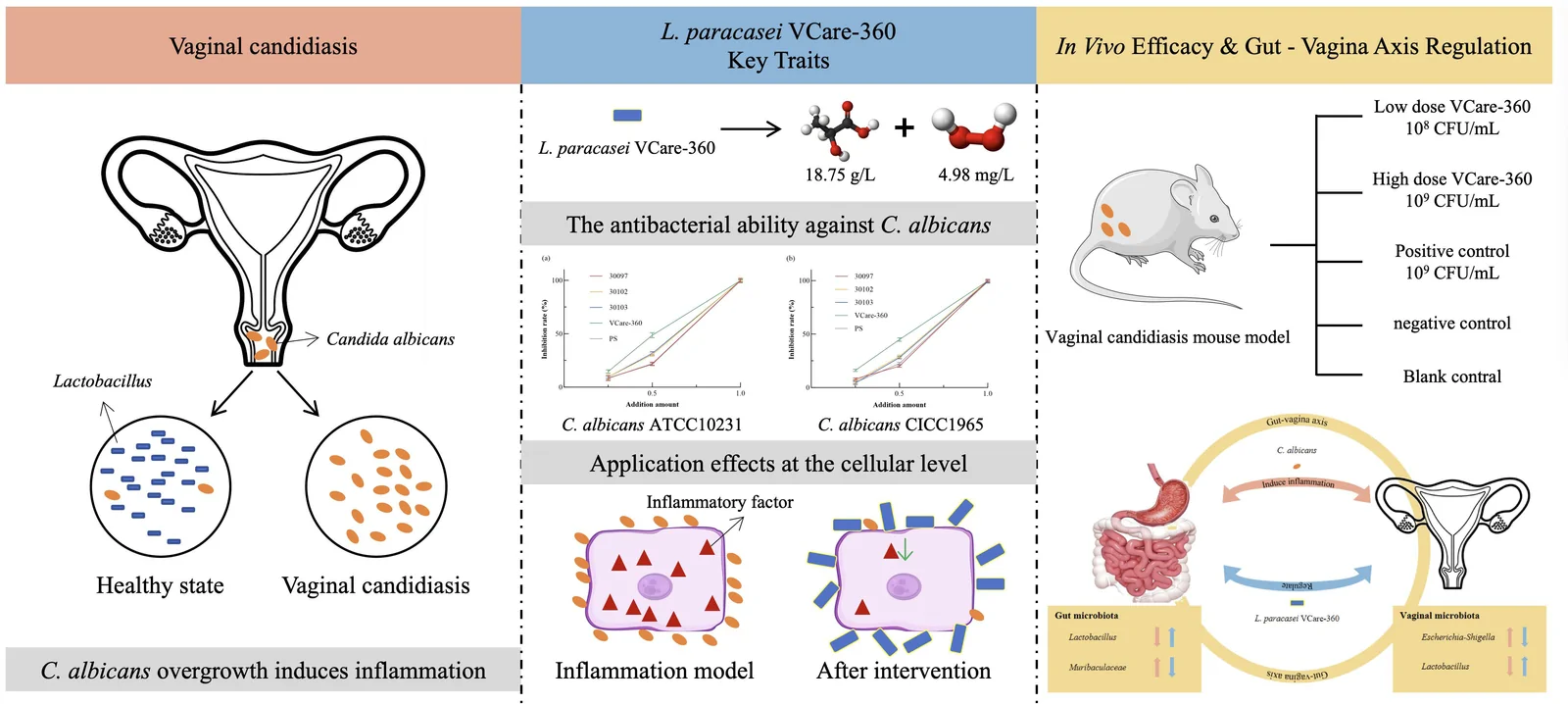

## 1 Introduction

Vulvovaginal candidiasis (VVC) is considered the second most common vaginal infection, which mainly caused by the opportunist fungus *Candida*, and it is estimated that about 75% of women have been infected at least once in their lifetime (Jessie and Lionakis, 2024). Fluconazole is currently the first-line antifungal agent for the treatment of VVC, with clinical studies reporting symptom relief and improved quality of life in approximately 96% of affected women. However, a substantial proportion of patients- reaching up to 63%- report persistent or recurrent symptoms following maintenance therapy (Satora et al., 2023). This high recurrence rate contributes to ongoing discomfort, psychological distress, and challenges in clinical management, raising concerns about the long-term efficacy of current antifungal strategies and the need for alternative or adjunctive therapies.

Fungal infections, particularly those caused by *C. albicans*, are a major cause of vulvovaginal inflammation and represent a significant burden in women’s health (San Juan Galán et al., 2023; Roselletti et al., 2023). Accumulating evidence indicates that *Latobacillus* species constitute the dominant microbial flora in the healthy vaginal ecosystem. *Lactobacillus* exerts its protective effects primarily through the production of various metabolites, including lactic acid, hydrogen peroxide, bacteriocins, and biosurfactants (Balakrishnan et al., 2022; Paniágua et al., 2021; Hefzy et al., 2021). Increasing attention has been directed toward probiotic *Lactobacillus* as a complementary or preventive intervention for VVC, demonstrating satisfactory efficacy in modulating the vaginal microbiota and reducing symptom recurrence (McKloud et al., 2021; Sun Z, et al., 2023; Ang et al., 2023). Spaggiari L et al. reported that VAGINNE^®^, a novel *Lactobacillus*-derived fermentation broth, effectively suppresses *C. albicans* growth, restores vaginal microbiome balance, and reduces inflammation and tissue damage in a murine VVC model (Spaggiari et al., 2022). Zhang J et al. reported that *L. gasseri* VHProbi E09 exhibits strong inhibitory activity against *Gardnerella vaginalis* and *C. albicans*, significantly reduces their adhesion to vaginal epithelial cells, and demonstrates additional antagonistic effects against enteropathogens, along with good gastrointestinal tolerance and intestinal adhesion (Zhang et al., 2024). Additionally, another study by Jang S J et al. reported that *L. fermentum* KBL674 significantly suppressed vaginal *C. albicans* infection and associated symptoms, including tissue thickness and immune cell infltration. This effect is achieved via direct or indirect inhibition and modulation of the gut microbiome composition, acting preventively (Jang et al., 2024). Accumulating evidence highlights a gut-vagina axis, where *Lactobacillus*-reactive immune cells migrate from the gut to the vagina, promoting the production of specific or cross-reactive antibodies and underscoring a systemic, coordinated defense mediated by the mucosal immune system (Amabebe and Anumba, 2020; Takada, 2025; Taghinezhad-S et al., 2021; Wei et al., 2023).

This study aimed to assess the anti-*Candida* activity of *L. paracasei* VCare-360 in human vaginal epithelial cells (VK2/E6E7) and its therapeutic potential in a murine model of *C. albicans*-induced vulvovaginal candidiasis. These results provide evidence for the use of probiotics to alleviate VVC caused by *C. albicans*.

## 2 Materials and methods

### 2.1 Strain preparation

*L. paracasei* VCare-360 (CGMCC 31095) and other *L. paracasei* strains used in this study were isolated from saccharification samples of a winery and maintained in our laboratory. The positive control strain (PS) was obtained from a commercially available women’s health probiotic product (Chr. Hansen China Co., Ltd.). *C. albicans* ATCC10231 and CICC1965 were obtained from the China Center of Industrial Culture Collection (Beijing, China). All *L. paracasei* strains and the PS strain were cultured in MRS broth at 37 °C for 18 h, while the *C. albicans* strains were cultured in YPD broth at 30 °C for 18 h.

### 2.2 Cell culture

Human epithelial cells VK2/E6E7 (ATCC CRL-2616) was obtained from GuangZhou Jennio biotech Co., Ltd, which were cultured in Keratinocyte Serum-free Medium (KSFM, Gibco, Carlsbad, CA, USA) supplemented with 10% fetal bovine serum (FBS, Gibco, Carlsbad, CA, USA), and antibiotics (10 U/mL ampicillin, 10 μg/mL streptomycin) at 37°C in a 5% CO_2_ humidified incubator.

### 2.3 Quantitative determination of hydrogen peroxide

According to the quantification method described by Marty-Teysset et al (Marty-Teysset et al., 2000), the concentrations of H_2_O_2_ were determined by measuring the amount of quinoneimine formed from the oxidation of 4-aminoantipyrine and phenol by hydrogen peroxide. To prepare the reaction mixture, 0.5 mL of fermentation supernatant, 3.75 mL of phosphate buffer solution (pH 7.0), 0.1 mL of water-saturated phenol solution, 0.15 mL of horseradish peroxidase solution (10 U/mL), and 0.5 mL of 4-aminoantipyrine solution (4 mg/mL) were combined. For the control group, the fermentation supernatant was replaced with an equivalent volume of phosphate buffer solution. The mixture was incubated at 37°C for 15 min. The absorbance was then measured at a wavelength of 505 nm.

### 2.4 Quantitative determination of lactic acid

Briefly, *Lactobacillus* supernatant with corresponding cultures for 18 h was collected at 12000 r/min for 2 min at 4°C. The lactic acid content was determined using an Agilent 1260 series high-performance liquid chromatography (HPLC, Agilent Technologies Inc., Santa Clara, CA, USA). The supernatant was analyzed using Bio-RadAminex HPX-87HColumn (300 mm×7.8 mm, 8 μm; DAD 210 nm). The mobile phase was 5 mM sulfuric acid solution. The HPLC calibration curve of lactic acid was calculated with 10 different concentrations of the standard. The R^2^ value of the standard curve was 0.9999 and the detection range was from 0 to 10 g/L.

### 2.5 Determination of the inhibitory activity

Briefly, *Lactobacillus* supernatant with corresponding cultures for 18 h was collected at 12000 r/min for 2 min at 4°C. The overnight culture of *C. albicans* in YPD was adjusted to 10^5^ CFU/mL, and 100 μL suspension was transferred to each well of 1 96-well plate, then 100 μL *Lactobacillus* supernatant was added (the addition amount of fermentation supernatant was 1). 100 μL MRS medium was used as the blank control, 100 μL G418 was used as the PS. The fermentation supernatant was diluted two-fold (the addition amount of fermentation supernatant was 0.5) and four-fold (the addition amount of fermentation supernatant was 0.25), and the same antibacterial assay was performed on these dilutions. The inhibition rate is calculated using the following formula: Inhibition Rate(%)=(OD_blank_-OD_sample_)/OD_blank_×100%. The IC_50_ value was calculated based on the inhibition effects at different concentrations.

### 2.6 Adhesion assay and adhesion of competition assay

The adhesion experiment was carried out according to the previous research method (Zhang et al., 2022; Chen et al., 2014). The VK2/E6E7 cells at a density of 1×10^6^ per well were cultured in 12-well cell culture plates with sterile cover glass placed in the holes beforehand at 37°C for 24 h. Discarding the medium, 1×10^8^ CFU/mL of *L. paracasei* VCare-360 and 1×10^7^ CFU/mL of *C. albicans* suspended in 2 mL KSMF were added, which were incubated 2 h at 37°C under 5% CO_2_. Following incubation, non-adherent probiotics were washed away with PBS buffer. The samples were fixed with methanol at room temperature for 20 min. After fixation, the fixative was removed by rinsing with ddH_2_O, and the samples were air-dried. Gram staining was performed using a commercial staining kit. The coverslips were then dried, mounted onto glass slides using neutral resin, and secured for microscopic observation. To quantify *C. albicans* adhesion to VK2/E6E7 cells, scanning electron microscopic examination was conducted, the experimental methods referred to the previous research method (Wang et al., 2018). Twenty cells were randomly selected, and the number of adherent *C. albicans* per cell was counted. The adhesion of competition assay was same as described above, with the only difference being the addition of *L. paracasei* VCare-360 at a concentration of 10^8^ CFU/mL versus a suspension of *C. albicans* at a concentration of 10^7^ CFU/mL.

### 2.7 *In vitro* anti-inflammatory activity screening of probiotics

The VK2/E6E7 cells at a density of 1×10^5^ per well were cultured in 12-well cell culture plates at 37°C for 24 h. Discarding the medium, which was replaced with antibiotic-free medium. The cells were stimulated with *C. albicans* at a final concentration of 10^5^ CFU/mL under 5% CO_2_, 37°C for 2 h. *L. paracasei* VCare-360 was diluted to a final concentration of 10^9^ CFU/mL using KSMF complete medium, then 10 μL dilution solution was added to the well, resulting in a final concentration of 10^7^ CFU/mL. The incubation group without pathogenic bacteria and probiotics was the blank control, and with pathogenic bacteria alone was the model group. After co-incubation at 5% CO_2_, 37°C for 6 h, the medium was removed, and the cells were washed with PBS for three times. The RNA of cells were extracted according to the steps of the cell RNA extraction kit. The expression levels of TNF-α, IL-8, IL-6, and IL-1β were detected by RNA detection kit. The list of primers was provided in the **Table 1**.

**Table 1:** Primers used in real-time PCR.

Gene	Forward	Reverse
TNF-α	GGTCCACTGTGCAAGAAGAGA	TCACCGCTTCAGAAAACCACC
IL-8	CTAGGACAAGAGCCAGGAAGAA	AACTGCACCTTCACACAGAGC
IL-6	TCTGCCAGTGCCTCTTTGC	AGCCCTGAGAAAGGAGACATGTA
IL-1β	ATGATGGCTTATTACAGTGGCAA	GTCGGAGATTCGTAGCTGGA

### 2.8 Animals test

Seven-week-old female BALB/c mice (18.0-21.0 g) were obtained from Beijing Vital River Laboratory Animal Technology Co., Ltd. The mice were kept at 22-24°C and 50 ± 10% humidity with a 12-h light/dark cycle, and they were fed standard laboratory chow and allowed water ad libitum. All experiments were carried out in accordance to Jiangsu Institute of Parasitic Diseases guidelines for the Care and Use of Laboratory Animals and were approved by the Committee for the Care and Use of Laboratory Animals at Jiangsu Institute of Parasitic Diseases (IACUC-JIPD-2021043). The mice were randomly divided into six groups as shown in **Figure 1**, VCare-360 (a) low dose (10^8^ CFU/mL) group, (b) high dose (10^9^ CFU/mL) group, (c) positive control (10^9^ CFU/mL), (d) negative control (model group), (e) blank control group. The fungal vaginitis of model was established according to the method described previously (Liao et al., 2017). The animals received subcutaneous injection of 0.4 mg/mice of estradiol valerate (E129414–250 mg, aladdin) diluted in 100 μL of sesame oil 3 days before fungal inoculation (day -3) and on the day of infection (day 0). Infection of mice was performed by intravaginal inoculation of 20 μL of *C. albicans* (10^10^ CFU/mL) and lasted for 5 days. The treatment started 48 h after infection, 200 μL of different concentration VCare-360 was intragastric administration in experimental mice once a day for 12 days from day 5 till day 17. Mice in the blank control group were intragastric administration with 200 μL phosphate buffer solution (PBS) and mice in the PS were intragastric administration with 200 μL market competing products once a day over the same period. The blank control group, no operation was performed. On day 18, vaginal lavage fluid was collected to detect the flora of vaginal tissue, and the fecal samples were collected for gut microbiota detection. On day 19, all experimental mice were killed. Body weight was monitored twice a week.

**Figure 1:**
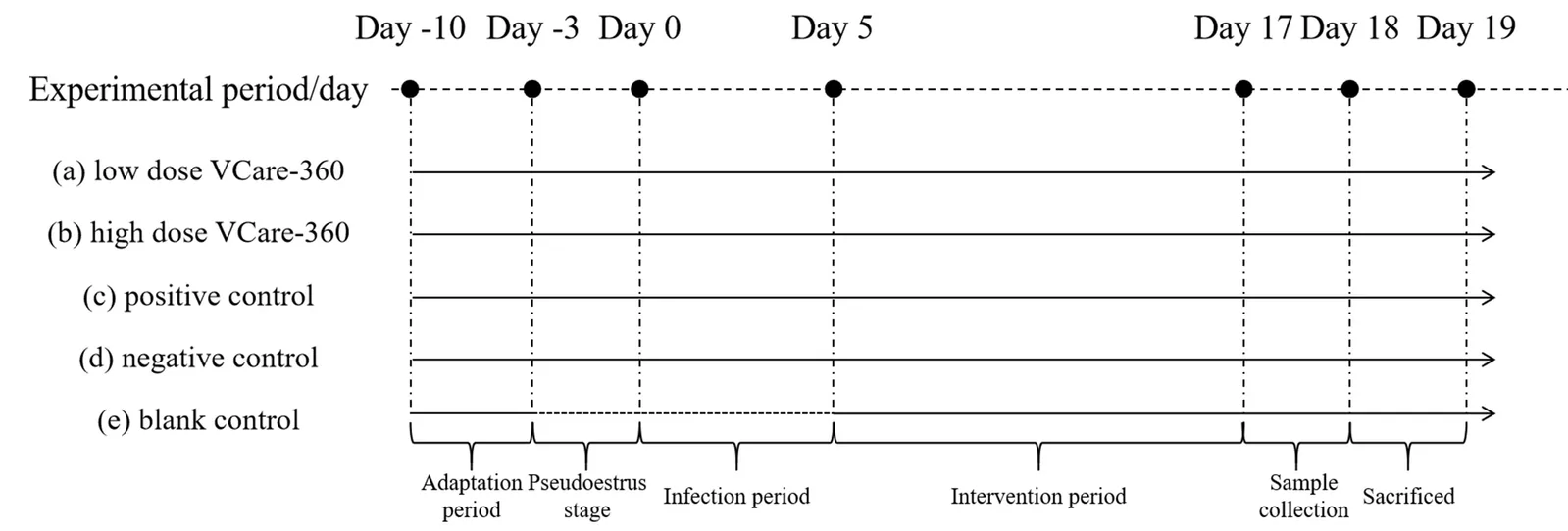
Experimental procedure used in the fungal vaginitis mouse model. The timeline illustrates the experimental design spanning from Day-10 to Day 19, divided into distinct phases: adaptation period (Day-10 to Day-3), pseudoestrus induction stage (Day-3 to Day 0), infection period (Day 0 to Day 5), intervention period (Day 5 to Day 17), sample collection (Day 17 to Day 18), and sacrifice (Day 19). Five experimental groups were included: **(a)** low-dose VCare-360 treatment group, **(b)** high-dose VCare-360 treatment group, **(c)** positive control group (infected mice receiving standard antifungal therapy), **(d)** negative control group (infected mice without therapeutic intervention), and **(e)** blank control group (uninfected mice administered vehicle only). Solid circles on the timeline denote key experimental timepoints for procedural milestones. All groups underwent synchronized protocols during the adaptation, pseudoestrus, and infection phases, with differential interventions applied during the intervention period based on group assignment.

### 2.9 Measurement of vaginal epithelial cell exfoliation

Referring to the method of Yano et al (Yano et al., 2011), a sample of 10 μL from the vaginal lavage fluid of mice collected on day 17 was transferred to a slide and gently applied with the outer wall of the 10 μL gun head. After the specimens were fixed with fixative, Diff-Quik staining was performed according to the kit instructions. The number of epithelial cells shed in each of the five microscopic fields was randomly counted under a light microscope at 400 magnification.

### 2.10 Microbial quantification by quantitative polymerase chain reaction

The total DNA of mouse vaginal lavage fluid was extracted using the FastDNA Spin Kit for soil and QIAick Gel extraction kit. The ChamQ Universal SYBR qPCR Master Mix (Yisheng Biotechnology (Shanghai) Co., Ltd, China) was employed to configure the fluorescence quantitative PCR reaction system (10 µL), composed of 5 µL of SYBR qPCR Master Mix, 0.5 µL of upstream primers, 0.5 µL of downstream primers, 3 µL of ddH_2_O, and 1 µL of DNA template. The RT-qPCR reaction program was as follows: 95°C for 30 s, 40 cycles (95°C for 5 s, 60°C for 30 s), and a dissolution curve from 65°C to 95°C, incremented by 0.1°C/s. Samples were determined to have reached the fluorsescence threshold cycle value (CT), and calculated the copy number based on the standard curve (Log copies/μL versus CT value). Each sample was tested in triplicate. In order to detect and quantify by qPCR the presence of *L. paracasei*, 1145-S primer pair was used, (5’-GCAGGATGCTGATTGTTAGCAG-3’ and 5’-CTCCGACCAAAGCGACTATGAC-3’), which amplifies a fragment of 500 bp of the 1145-S gene (Sun, L. H., et al., 2023).

### 2.11 Histopathological analysis of mouse vaginal tissue

The vaginal tissues were aseptically removed for the histopathological analysis, the samples were analyzed according to Araújo et al (Araújo et al., 2020). with modifications. The intensity of inflammation was determined through the evaluation of hematoxylin and eosin (H&E) stained samples in 10 fields at ×400 under a standard light microscope. The pathological changes of vaginal tissues, including inflammatory response, epithelial cell shedding, and invasion of *C. albicans* in hyphal or yeast form, were recorded and alalyzed.

### 2.12 Elisa

Vaginal tissue samples (20 mg) were placed in 200 μL of Radioimmunoprecipitation assay (RIPA) lysis buffer containing 2% (v/v) protease inhibitor cocktail and 2% (v/v) phosphatase inhibitor mixture (Beyotime Biotechnology (Shanghai) Co., Ltd, China). The tissue samples were then homogenized using a high-throughput tissue grinder, and then the homogenates were centrifuged at 12000 rpm for 15 min at 4°C. The supernatants were carefully collected for the resulting vaginal tissue and were utilized for the quantification of myeloperoxidase (MPO), interleukin-1β (IL-1β), interleukin-6 (IL-6), interleukin-10 (IL-10), interleukin-4 (IL-4), tumor necrosis factor α (TNF-α) levels in accordance with the manufacturer’s instructions provided within the assay kit. Blood was collected from mice via orbital sinus puncture and transferred into procoagulant-treted tubes. One mL was collected from each mouse. The blood was allowed to clot for 2 h at room temperature. Following coagulation, the samples were centrifuged at 3500 to 4000 rpm for 15 min at room temperature. The supernatant was transferred to a new sterile centrifuge tube, avoiding aspiration of the precipitated fraction, and were utilized for the quantification of interleukin-1β (IL-1β), interleukin-6 (IL-6), interleukin-10 (IL-10), interleukin-4 (IL-4), tumor necrosis factor α (TNF-α) levels in accordance with the manufacturer’s instructions provided within the assay kit.

### 2.13 Microbial community structure analysis

On day 18, the collected vaginal lavage fluid and fecal samples were immediately placed in liquid nitrogen and then stored at -80°C for subsequent analysis. The DNA was extracted with the FastDNA Spin Kit for soil, and the V3-V4 region of the bacterial 16 S rRNA gene was amplified by polymerase chain reaction using primers 341F and 806R. The amplicons from all samples were pooled and sequenced on the Illumina Nextseq 2000 platform.

Bioinformatics analysis was performed on the Majorbio I-Sanger Cloud Platform (http://www.i-sanger.com). ASVs were generated using DADA2, and taxonomy was assigned against the SILVA v138 database with a confidence threshold of 0.7. Chloroplast and mitochondrial sequences were removed. Alpha diversity (Chao1, Shannon, Simpson) was calculated using mothur. Differences in alpha diversity were tested using the Kruskal-Wallis test (multi-group) or Wilcoxon rank-sum test (two-group), with FDR correction. Beta diversity was assessed based on Bray-Curtis distance using PCoA, and group differences were evaluated by ANOSIM (999 permutations). All statistical analyses were conducted in R (v3.3.1).

### 2.14 Statistical analysis

For all assays, the results were the average of three independent experiments performed in triplicate, except for the mice test, which was evaluated in six independent experiments. Statistical analysis was done using the GraphPad software and the statistical tests used for each assay are indicated on their respective results. Results were considered statistically different for at least *p* < 0.05.

## 3 Results

### 3.1 Hydrogen peroxide, lactic acid production and the inhibitory activity

Four strains of *L. paracasei* were selected for *in vitro* metabolite detection. The results (**Table 2**) showed that *L. paracasei* VCare-360 exhibited high yields of hydrogen peroxide and lactic acid, at 4.98 mg/L and 18.75 g/L respectively. What’s more, the inhibitory activity of *L. paracasei* VCare-360 was evaluated at different concentrations against *C. albicans*. The suspension inhibited 99.69% of the fungus growth. The concentration of the suspension that inhibited 50% of *C. albicans* growth (IC_50_) was 0.54 (**Figure 2**).

**Table 2:** Lactic acid and H_2_O_2_ production of by *Lactobacillus* and its antifungal activity against *C. albicans.*.

*Lactobacillus* strain	H_2_O_2_ (mg l^-1^)	Lactic acid (g l^-1^)	Inhibition rate (%)		IC_50_ (the addition amount of fermentation supernatant)	
			ATCC10231	CICC1965	ATCC10231	CICC1965
*L. paracasei* 30097	1.95 ± 0.08	16.62 ± 0.66	99.91 ± 1.66	100.01 ± 1.11	0.70	0.71
*L. paracasei* 30102	1.67 ± 0.07	16.71 ± 0.67	99.88 ± 1.35	99.88 ± 1.03	0.65	0.66
*L. paracasei* 30103	4.78 ± 0.11	15.44 ± 0.62	99.64 ± 1.49	99.17 ± 1.01	0.65	0.67
*L. paracasei* VCare-360	4.98 ± 0.12	18.75 ± 0.56	99.82 ± 1.05	99.69 ± 1.28	0.51	0.54
PS	–	16.18 ± 0.83	99.58 ± 0.76	98.81 ± 1.30	0.69	0.70

**Figure 2:**
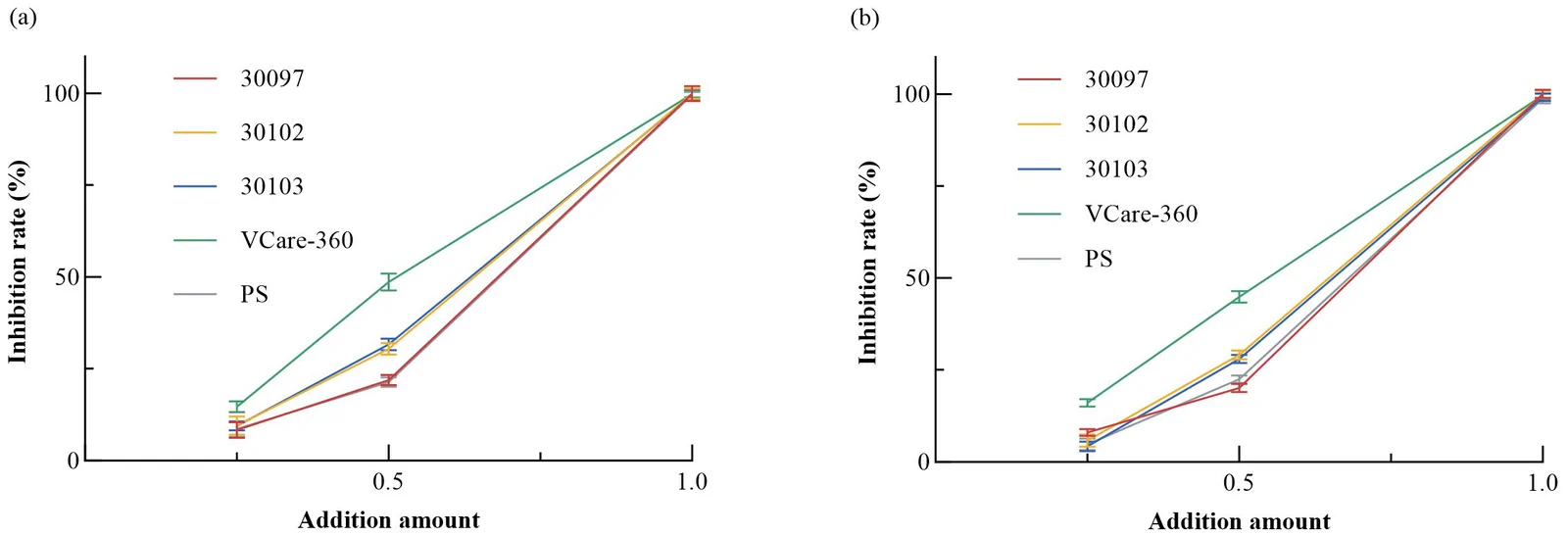
Inhibitory effects of *Lactobacillus* strains and controls on *C. albicans* growth. The graphs depict the inhibition rate (%) as a function of the addition amount for different test substances against two strains of *C. albicans*. **(a)** Inhibition curves against the opportunistic pathogen *C. albicans* ATCC 10231. **(b)** Inhibition curves against the opportunistic pathogen *C. albicans* CICC 1965. The tested groups include three *Lactobacillus* strains (30097, 30102, and 30103), VCare-360, and PS. Data points represent the mean ± standard deviation (SD). The IC_50_ values were calculated based on the inhibition rates measured at varying addition amounts.

The above *in vitro* results suggest that *L. paracasei* VCare-360 may possess excellent potential for inhibiting *C. albicans*, and thus it was selected as the strain for subsequent experiments.

### 3.2 Adhesion assay and effects of *Lactobacillus* on pathogen adhesion

Adhesion plays a key role in the beneficial effects of vaginal *Lactobacillus*, and is also the key virulence factor for pathogens such as *C. albicans*, *Gardnerella*, *Atopobium* (He et al., 2020). Both *L. paracasei* VCare-360, *C. albicans*, PS were able to adhere, to different degrees, to VK2/E6E7 cells (**Figures 3a–e**). The adhesion capacity of L. paracasei VCare-360 to VK2/E6E7 cells was significantly superior to that of the PS strain. Specifically, the adhesion index for VCare-360 was 19.85 ± 5.85 CFU/cell, compared to 8.25 ± 3.16 CFU/cell for PS (*p* < 0.0001). To evaluate the antagonistic effect of Lactobacillus strains against C. albicans adhesion to VK2/E6E7 cells, a co-culture assay was performed. As shown in **Figures 3f–h**, L. paracasei VCare-360 exhibited a potent inhibitory activity. The adhesion index of C. albicans in the control group was 41.75 ± 13.14 CFU/cell. Co-culture with PS significantly reduced this index to 13.45 ± 3.71 CFU/cell (*p* < 0.0001). Notably, L. paracasei VCare-360 demonstrated an even stronger inhibition, reducing the adhesion index to 10.95 ± 4.46 CFU/cell (*p* < 0.0001 vs. control).

**Figure 3:**
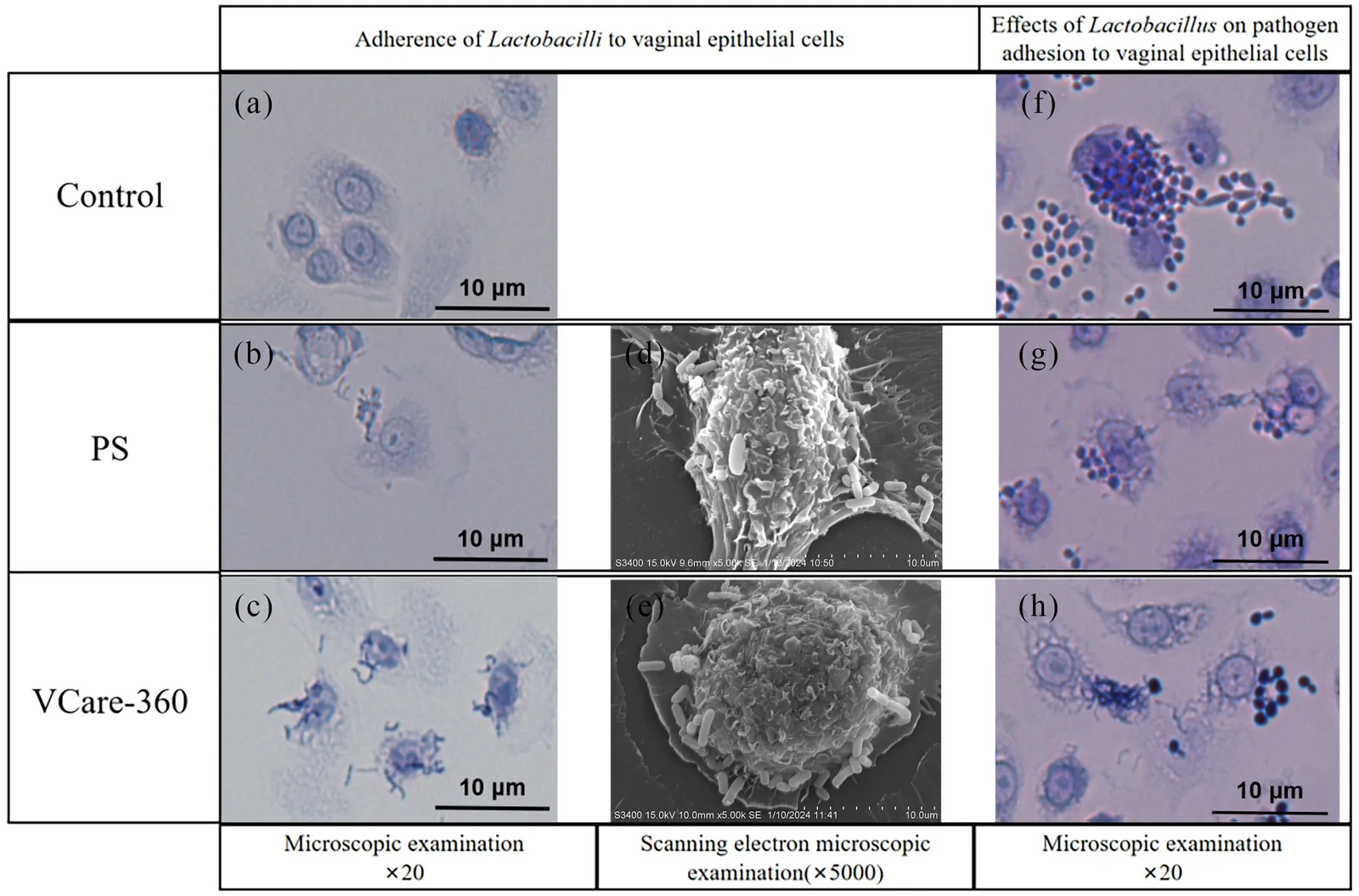
Adhesion of *Lactobacillus* strains to VK2/E6E7 vaginal epithelial cells and their effect on pathogen adhesion. The figure presents microscopic and scanning electron microscopic (SEM) analyses of bacterial interactions with host cells. **(a–c)** Light microscopy images (20 × magnification) showing the adherence of *Lactobacillus* to VK2/E6E7 cells in different groups: **(a)** Control group (untreated cells), **(b)** PS group (positive control), and **(c)** VCare-360 group. **(d, e)** SEM images (5000 × magnification) providing a high-resolution view of the morphological details of *Lactobacillus* adherence to the surface of VK2/E6E7 cells in the **(d)** PS group and **(e)** VCare-360 group. **(f–h)** Light microscopy images (20 × magnification) illustrating the competitive adhesion assay between *Lactobacillus* and *C. albicans* on VK2/E6E7 cells: **(f)** Control group showing extensive pathogen colonization, **(g)** PS group, and **(h)** VCare-360 group demonstrating the inhibitory effect of the treatment on pathogen attachment. Scale bars represent 10 μm.

### 3.3 Inflammatory gene expression responses by VK2/E6E7 cells to *C. albicans* are influenced by *L. paracasei* VCare-360 and PS

VK2/E6E7 cells were initially stimulated with *C. albicans* at a multiplicity of infection (MOI) of 1 for 2 h, followed by co-culture with probiotic strains at an MOI of 100 for 6 h. Four experimental groups were established, control group (unstimulated cells, control), *C. albicans* challenge group (model), positive probiotic control group (PS), and *L. paracasei* VCare-360 group (VCare-360). After incubation, total RNA was extracted from the cell, and real-time RT-PCR panels were used to measure differences in mRNA expression of microbial receptors and genes involved in intracellular signal transduction. *C.albicans* infection significantly VK2/E6E7 cell mRNA expression of the pro-inflammatory cytokine gene TNF-α, IL-6, IL-8. Results (**Figure 4**) demonstrated that positive probiotic and *L. paracasei* VCare-360 exhibited superior anti-inflammatory efficacy compared to controls, with statistically significant reductions in cytokine expression (*p* < 0.05). Furthermore, the *L. paracasei* VCare-360 groups showed more stable and reproducible outcomes across experimental replicates.

**Figure 4:**
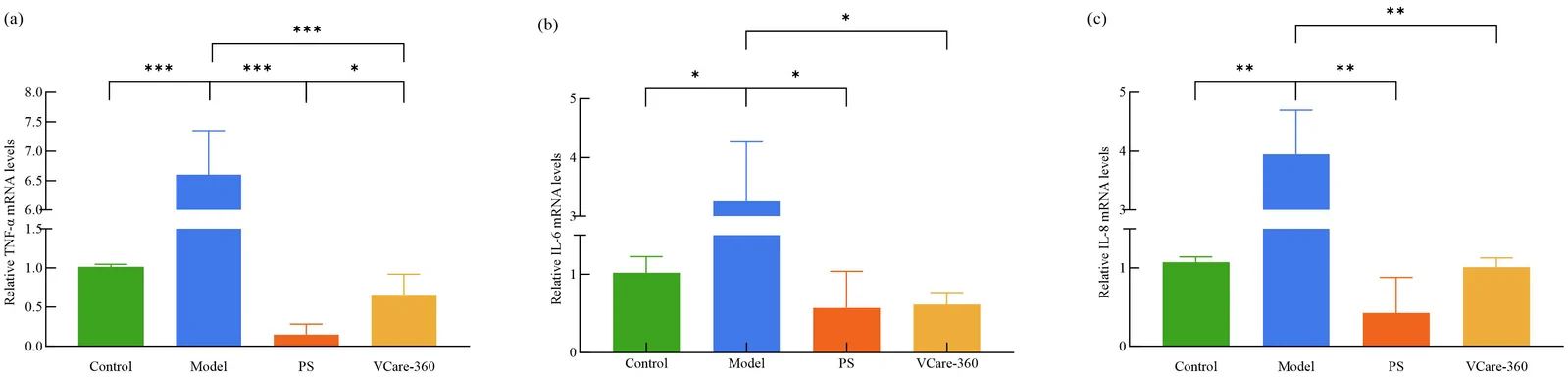
Effects of VCare-360 on the mRNA expression levels of inflammatory cytokines in VK2/E6E7 cells. The bar charts display the relative mRNA expression levels of **(a)** tumor necrosis factor-alpha (TNF-α), **(b)** interleukin-6 (IL-6), and **(c)** interleukin-8 (IL-8) across four experimental groups: Control, Model, PS, and VCare-360. Data are presented as mean± standard deviation (SD). Statistical significance is indicated by asterisks: *p<0.05, **p<0.01, and ***p<0.001 compared to the Model group or PS group.

### 3.4 Effect of *L. paracasei VCare-360* on body weight changes in mice

Body weights were continuously monitored to evaluate the general health status of the mice. Two-way ANOVA revealed a significant interaction between time and treatment groups (*p* < 0.0001), indicating that the effect of the intervention on body weight varied over time. Compared to the Control group (final mean: 20.28 ± 0.60 g), all infected groups exhibited transient weight loss following infection. Specifically, at Day 3 of intervention, the Model group reached its nadir with a mean body weight of 17.99 ± 0.90 g, which was significantly lower than that of the Control group (20.34 ± 0.60 g). However, intervention with *L. paracasei VCare-360* mitigated this weight loss trend. Mice in the treatment groups (*L. paracasei VCare-360-*L/H) showed a progressive recovery in body weight throughout the intervention period. By the endpoint, the *L. paracasei VCare-360*-L group achieved a mean weight of 19.75 ± 1.10 g. Although full recovery to the baseline of the uninfected control was not completely achieved within the observation window, the data suggests that *L. paracasei VCare-360* administration helps alleviate the physiological stress caused by *C. albicans* infection and supports health restoration.

### 3.5 Evaluation of the *L. paracasei* VCare-360 colonization in mice with *Candida vaginitis*

To study the role of *L. paracasei* VCare-360 in the mice with vaginal *C.albicans* infection, the mice under pseudoestrus were inoculated with *C.albicans*, treated with *L. paracasei* VCare-360 and take samples on the 17^th^ day after inoculation. To analyze the colonization of *L. paracasei* VCare-360, we measured the loads of *L. paracasei* VCare-360 in the vaginal lavage fluid on days 17 by qPCR. *L. paracasei* was not detected *in vivo* prior to intervention. The detection of *L. paracasei* VCare-360 colonization on day 17, suggests that its presence may exert a certain inhibitory effect on the *in vivo* growth of *C.albicans*.

### 3.6 Effect of *L. paracasei* VCare-360 on vaginal epithelial shedding

One of the key clinical features of fungal vaginitis, particularly *Candida*-associated vulvovaginal candidiasis (VVC), is the disruption of vaginal epithelial integrity, manifested as increased desquamation or shedding of epithelial cells. In the present study, we assessed the shedding of vaginal epithelial cells in mice on day 17 to determine whether *L. paracasei* VCare-360 can affect epithelial exfoliation induced by *C. albicans*. As shown in **Figure 5**, approximately 50.7 cell per field were counted in the model group, while 17.0 cells per field were counted in the control group. *L. paracasei* VCare-360 in the high dose significantly alleviated epithelial exfoliation compared to the model group (*p* < 0.05). in the present experiment, different doses of *L. paracasei* VCare-360 had a certain inhibitory effect on vaginal epithelial cell shedding, indicating that it was effective in preventing the spread and adhesion of pathogens in tissues. Competitor group showed significantly alleviated epithelial exfoliation compared to the model group (*p* < 0.01).

**Figure 5:**
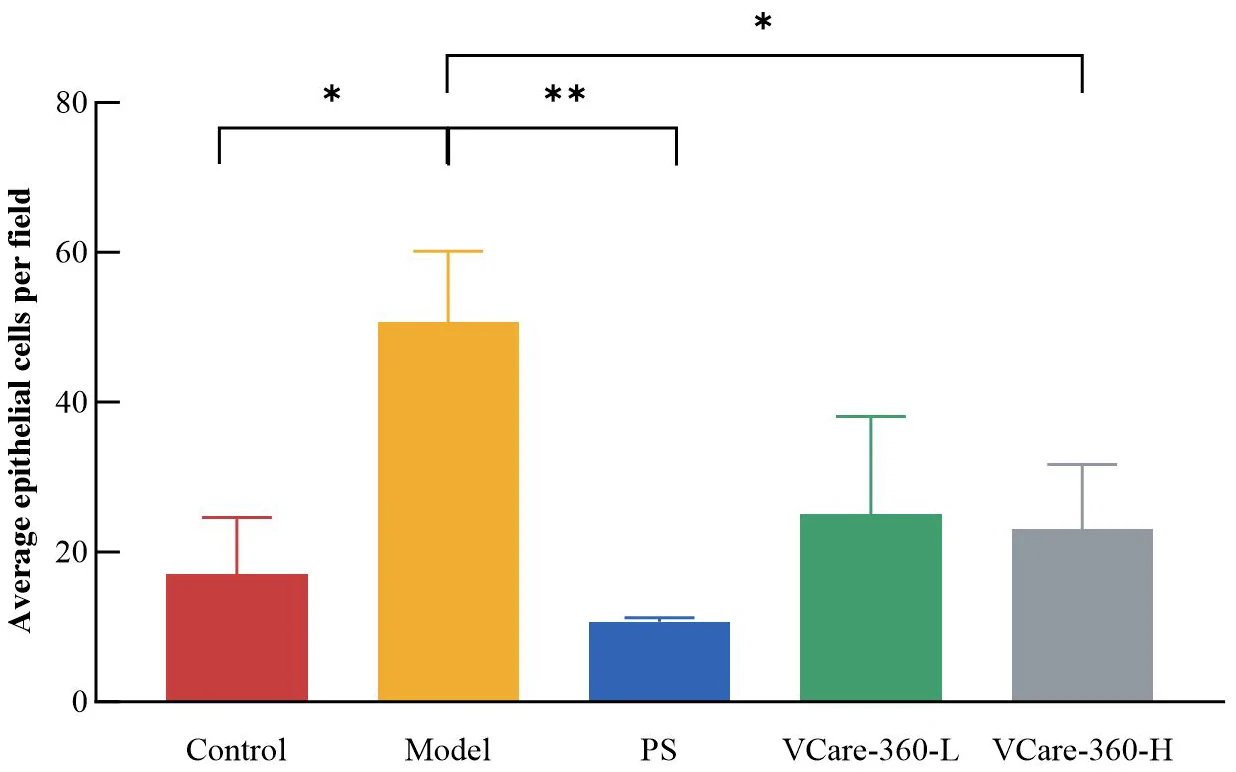
Effect of *L. paracasei* VCare-360 on *C. albicans*-induced epithelial cell exfoliation. The bar chart quantifies the average number of exfoliated epithelial cells per field across different experimental groups: Control, Model, PS (positive control), VCare-360-L (low dose), and VCare-360-H (high dose). Data are presented as mean ± standard deviation (SD). Statistical significance compared to the Model group is indicated by asterisks: *p<0.05, **p<0.01.

### 3.7 Histopathological analysis of mice vaginas

Histological examination revealed distinct morphological changes in vaginal tissues across groups (**Figure 6**). The Control group exhibited a smooth, continuous vaginal epithelium with an intact tissue structure and no inflammatory infiltration (**Figure 6a**, black arrow). In contrast, the Model group displayed severe pathological damage, characterized by discontinuous mucosal epithelium, superficial erosion, and micro-defects, accompanied by extensive local inflammatory cell infiltration in the mucosa (**Figure 6b**, red and blue arrows).

**Figure 6:**
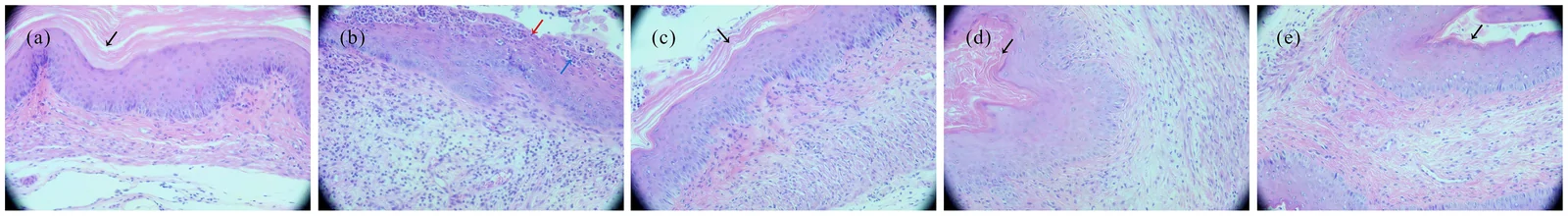
Histological analysis of vaginal tissues using H&E staining. Representative photomicrographs show the morphological changes in vaginal tissue sections across different experimental groups: **(a)** Control, **(b)** Model, **(c)** PS (positive control), **(d)** VCare-360-L (low dose), and **(e)** VCare-360-H (high dose). In the Control group **(a)**, the vaginal epithelium appears intact and smooth with well-organized layers (black arrow). In contrast, the Model group **(b)** exhibits severe tissue damage, characterized by superficial erosion, structural defects (red arrow), and significant local infiltration of inflammatory cells into the vaginal mucosa (blue arrow). Treatment with PS **(c)**, VCare-360-L **(d)**, and VCare-360-H **(e)** alleviates these pathological changes, showing restored epithelial integrity and reduced inflammatory infiltration compared to the Model group. Black arrows indicate smooth vaginal epithelium; blue arrows indicate local inflammatory cell infiltration; red arrows indicate superficial erosion and holes.

Probiotic intervention significantly alleviated these symptoms. The PS group showed partial recovery of the epithelial layer (**Figure 6c**). Notably, the VCare-360-L (**Figure 6d**) and VCare-360-H (**Figure 6e**) groups demonstrated a marked restoration of vaginal architecture. In these treatment groups, the epithelial cells were tightly packed with uniform nuclei and consistent eosinophilic staining, indicating restored cellular metabolic activity. Furthermore, the underlying stroma showed reduced edema and no obvious fibrous hyperplasia, suggesting that *L. paracasei* VCare-360 effectively promotes mucosal recovery and alleviates inflammation.

### 3.8 Effects of *L. paracasei* VCare-360 on the expression of cytokines and myeloperoxidase after by *C. albicans* infection

To evaluate the systemic immunomodulatory effects of *L. paracasei* VCare-360, cytokine levels and MPO activity were measured in serum on day 17 post-infection (**Figure 7**). Compared to the Model group, intervention with *L. paracasei* VCare-360 significantly promoted an anti-inflammatory response. Specifically, the levels of the anti-inflammatory cytokine IL-10 were significantly elevated in all treatment groups (PS, VCare-360-L, and VCare-360-H; *p* < 0.01). Additionally, IL-4 levels were significantly increased in the PS and VCare-360-H groups (*p* < 0.05 or *p* < 0.01), while the VCare-360-L group showed a non-significant upward trend. Conversely, the pro-inflammatory profile was effectively suppressed. The high dose of VCare-360 and PS treatment significantly reduced the levels of TNF-α, IL-6, and MPO compared to the Model group (*p* < 0.0001 for TNF-α and MPO; *p* < 0.001 for IL-6). Although IL-1β levels also decreased in the treatment groups, the difference did not reach statistical significance compared to the Model group. These results indicate that *L. paracasei* VCare-360 effectively modulates the systemic immune balance by enhancing anti-inflammatory cytokines and suppressing key pro-inflammatory mediators.

**Figure 7:**
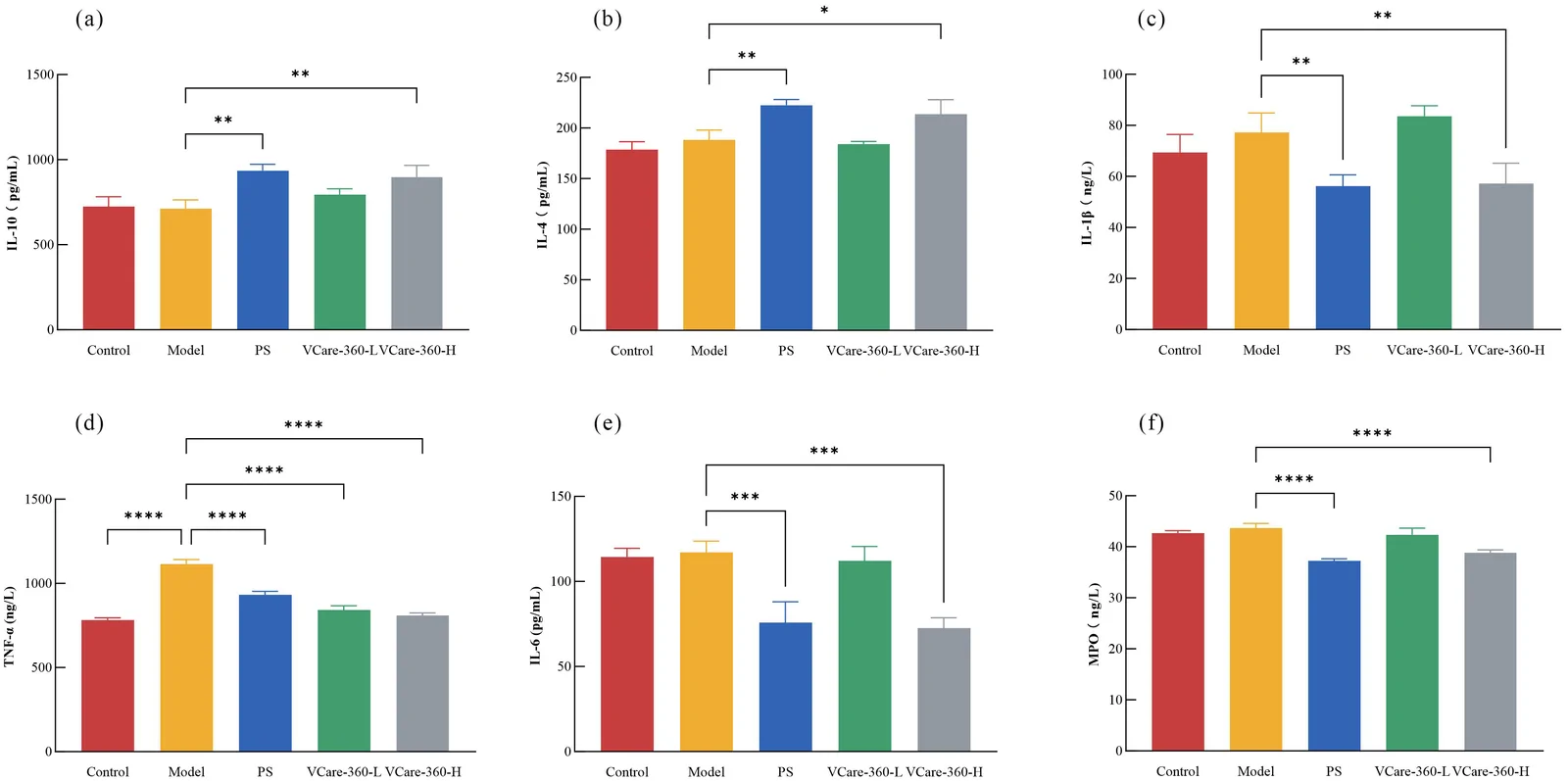
Effects of *L. paracasei* VCare-360 on serum inflammatory cytokine levels. The bar charts illustrate the concentrations of various inflammatory markers in serum across the experimental groups: Control, Model, PS (positive control), VCare-360-L (low dose), and VCare-360-H (high dose). The specific markers analyzed are **(a)** interleukin-10 (IL-10), **(b)** interleukin-4 (IL-4), **(c)** interleukin-1β (IL-1β), **(d)** tumor necrosis factor-alpha (TNF-α), **(e)** interleukin-6 (IL-6), and **(f)** myeloperoxidase (MPO). Data are presented as mean ± standard deviation (SD). Statistical significance compared to the Model group is indicated by asterisks: *p < 0.05, **p < 0.01, ***p < 0.001, ****p < 0.0001.

Consistent with the serological findings, local immune responses in vaginal tissues were also significantly modulated by the probiotic intervention (**Figure 8**). Treatment with *L. paracasei* VCare-360 (both low and high doses) and PS led to a significant increase in IL-10 levels compared to the Model group (*p* < 0.05 or *p* < 0.01). Similarly, IL-4 expression was significantly upregulated in the PS and VCare-360-H groups (*p* < 0.05 or *p* < 0.01). Regarding pro-inflammatory factors, TNF-α levels were significantly downregulated across all intervention groups, including the Control, PS, and both VCare-360 dosage groups (*p* < 0.05 or *p* < 0.01 vs. Model). Furthermore, the high dose of VCare-360 significantly reduced IL-6 levels (*p* < 0.001) and MPO activity (*p* < 0.05) in vaginal tissues, indicating a reduction in neutrophil infiltration and local inflammation. While IL-1β levels showed a decreasing trend in the PS and VCare-360 groups, the changes were less pronounced than those of TNF-α and IL-6. Collectively, these data demonstrate that *L. paracasei* VCare-360 alleviates vaginal inflammation by shifting the local cytokine milieu toward an anti-inflammatory state and inhibiting neutrophil recruitment.

**Figure 8:**
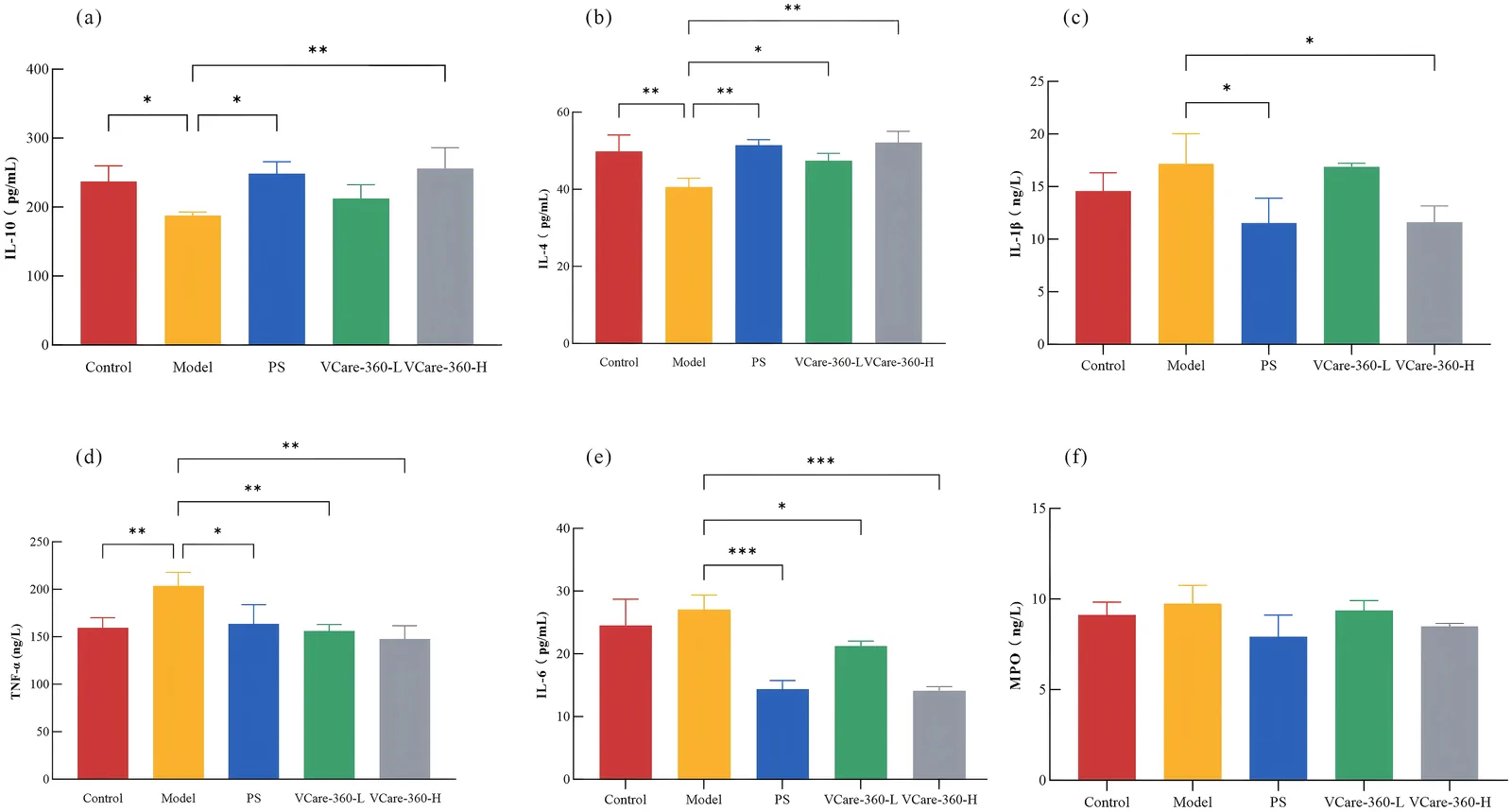
Effects of *L. paracasei* VCare-360 on inflammatory cytokine levels in vaginal tissue. The bar charts display the concentrations of inflammatory markers measured in vaginal tissue homogenates across the experimental groups: Control, Model, PS (positive control), VCare-360-L (low dose), and VCare-360-H (high dose). The specific markers analyzed are **(a)** interleukin-10 (IL-10), **(b)** interleukin-4 (IL-4), **(c)** interleukin-1β (IL-1β), **(d)** tumor necrosis factor-alpha (TNF-α), **(e)** interleukin-6 (IL-6), and **(f)** myeloperoxidase (MPO). Data are presented as mean ± standard deviation (SD). Statistical significance compared to the Model group is indicated by asterisks: *p < 0.05, **p < 0.01, ***p < 0.001.

### 3.9 Vaginal and fecal microbiota

A balanced vaginal microbiota is pivotal for maintaining barrier integrity, yet *C. albicans* infection often disrupts this microecological equilibrium. To evaluate the impact of *L. paracasei* VCare-360 on ecological stability, we analyzed α-diversity indices relative to the healthy control (**Figure 9**). Infection with *C. albicans* significantly altered the vaginal microecology in the Model group compared to the Control group. Specifically, species richness (Chao index) was markedly reduced (*p* < 0.01), while both the Simpson and Shannon indices were significantly elevated (*p* < 0.001 and *p* < 0.001, respectively). This suggests a loss of dominant beneficial taxa and an increase in community evenness driven by pathogen overgrowth. Following probiotic intervention, the dysbiotic trends showed signs of mitigation, although the microbiota did not fully return to the baseline levels of the Control group. Notably, the high-dose group (VCare-360-H) exhibited a partial recovery in species richness (Chao index), showing a significant difference from the Control (*p* < 0.05) but with a median value higher than that of the Model group. Similarly, while the Simpson and Shannon indices remained significantly different from the Control in all treatment groups (*p* < 0.01 or *p* < 0.001), the values in the VCare-360 treated groups tended to decrease compared to the Model group. These findings indicate that while L. paracasei VCare-360 intervention helps modulate the vaginal microbiota structure toward a healthier state, the ecological balance remains distinct from that of uninfected healthy controls within the observed timeframe.

**Figure 9:**
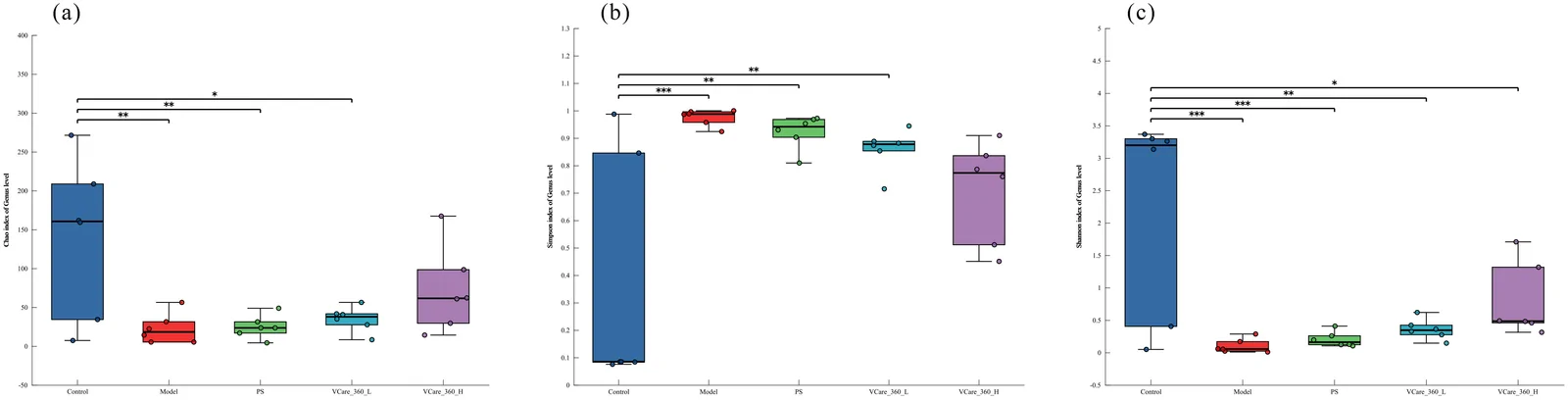
Analysis of vaginal microbiota alpha diversity. Box plots illustrate the differences in microbial community diversity across the experimental groups: Control, Model, PS (positive control), VCare-360-L (low dose), and VCare-360-H (high dose). The diversity was assessed using three indices: **(a)** Chao index, reflecting species richness; **(b)** Simpson index, reflecting species dominance and evenness; and **(c)** Shannon index, reflecting both species richness and evenness. Data are presented as box plots showing the median, interquartile range, and whiskers. Statistical significance compared to the Control group is indicated by asterisks: *p < 0.05, **p < 0.01, ***p < 0.001.

To evaluate the overall structural shifts in the vaginal microbiota, we performed Principal Coordinate Analysis (PCoA) based on unweighted UniFrac distances at the ASV level (**Figure 10**). The PCoA plot revealed distinct clustering patterns among the groups. The Model group formed a separate cluster significantly distant from the Control group along the PC1 axis, indicating that *C. albicans* infection profoundly disrupted the native vaginal microbial community structure. This separation was statistically validated by PERMANOVA analysis (*R* = 0.1384, *p* = 0.008), confirming significant compositional differences between the healthy and infected states. Following intervention, the treatment groups exhibited varying degrees of structural restoration. Notably, the *L. paracasei* VCare-360 groups (particularly the high-dose group) showed a trend of shifting away from the Model cluster and moving closer to the Control or PS clusters. The relatively tight distribution of samples within the VCare-360 groups suggests that the probiotic intervention helped stabilize the microbial community, reducing the heterogeneity caused by fungal infection and promoting a recovery toward a healthier ecological state.

**Figure 10:**
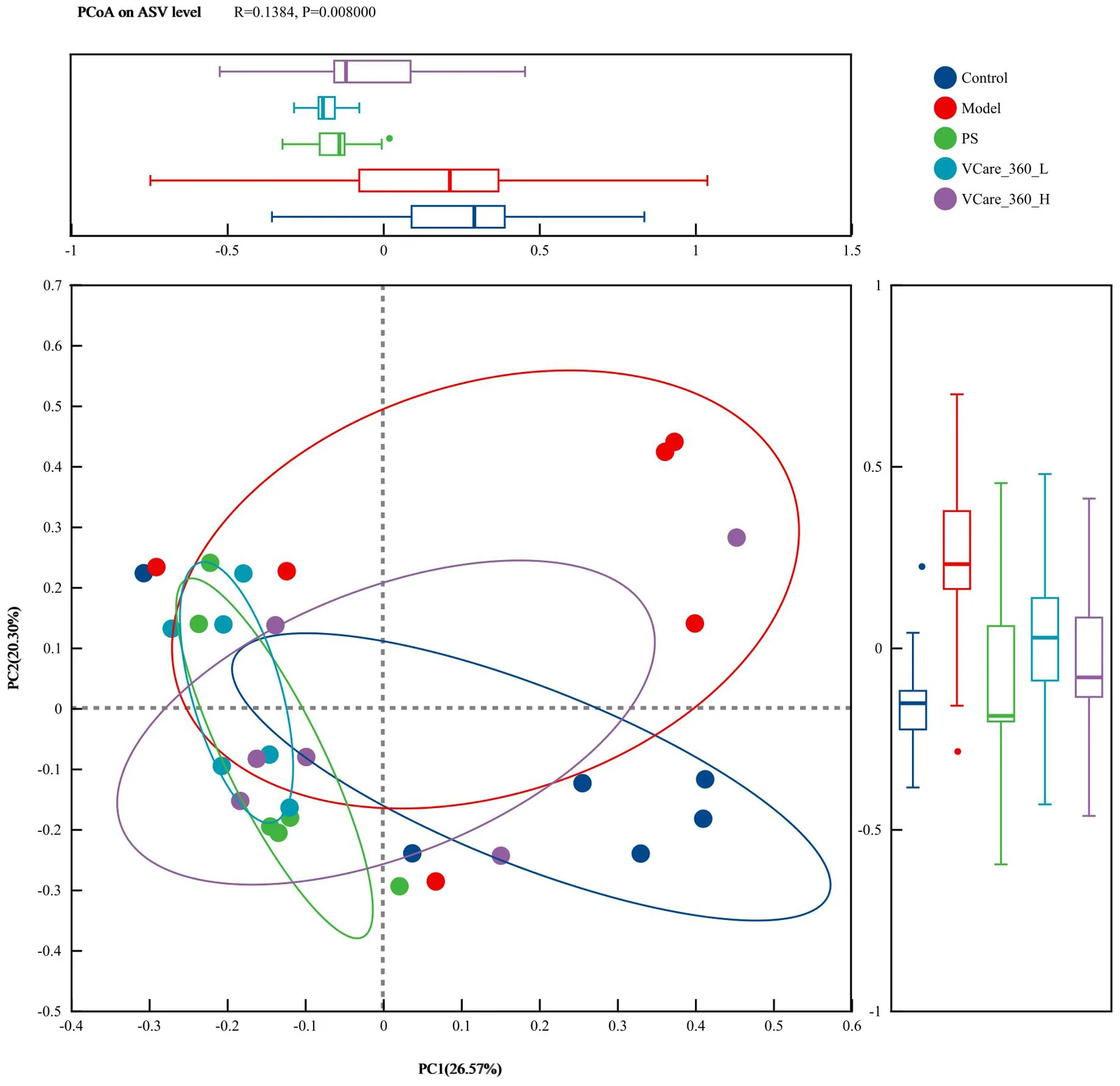
Analysis of beta diversity of the vaginal microbiota based on PCoA. Principal Coordinate Analysis (PCoA) plot showing the dissimilarity of microbial community structure among different groups at the ASV level. Each point represents an individual sample, and the ellipses represent the 95% confidence intervals for each group. The percentages on the axes (PC1 and PC2) indicate the proportion of variation explained by each principal coordinate. The box plots on the top and right sides display the distribution of samples along the PC1 and PC2 axes, respectively. The statistical significance of the separation between groups was determined by ANOSIM analysis (indicated by R and P values in the top left corner; R = 0.1384, P = 0.008). Different colors represent the following groups: Control (dark blue), Model (red), PS (green), VCare_360_L (cyan), and VCare_360_H (purple).

To comprehensively evaluate the impact of *L. paracasei* VCare-360 intervention on host microecology, we analyzed the taxonomic composition and identified significant biomarkers in both vaginal (**Figure 11**) and fecal (**Figure 12**) samples using Linear Discriminant Analysis Effect Size (LEfSe). At the phylum level (**Figure 11a**), the vaginal microbiota was predominantly composed of Firmicutes across all groups. However, distinct shifts were observed at the genus level (**Figure 11b**). To statistically validate these visual differences, LEfSe was employed with a threshold of LDA score > 4.0 (**Figures 11c, d**). The analysis revealed that the Model group was significantly enriched with opportunistic pathogens such as *Escherichia*-*Shigella* and *Enterobacteriaceae*, indicating dysbiosis. In contrast, the Control group showed a significant enrichment of *Muribaculaceae*. Notably, the high-dose VCare-360 group exhibited a unique signature characterized by high LDA scores for *Morganellaceae* and *Proteus*. While typically recognized as opportunistic, their emergence here likely represents a “transitional state” or specific response to the High-dose intervention, distinguishing this group from the Model group. The Low-dose group did not show distinct biomarkers compared to others, suggesting a milder modulation effect. Similarly, fecal microbiota analysis revealed that Firmicutes and Bacteroidetes were the dominant phyla across all groups (**Figure 12a**). *C. albicans* infection (Model group) induced a shift characterized by decreased Firmicutes and increased Bacteroidota abundance compared to the Control group. Probiotic intervention effectively reversed this trend; specifically, the Low-dose VCare-360 group restored the Firmicutes/Bacteroidota ratio to levels comparable to the Control group. At the genus level, LEfSe analysis (**Figures 12c, d**) confirmed significant alterations, with a threshold of LDA score >3.0. The Model group showed a marked depletion of *Lactobacillus* and an abnormal enrichment of *Muribaculaceae* compared to the Control group. Following intervention, the abundance of *Lactobacillus* was significantly restored in the treatment groups (VCare360-L: 20.34%; PS: 20.77%) compared to the Model group (10.23%). Furthermore, the aberrant increase of *Muribaculaceae* observed in the Model group was gradually normalized in the probiotic-treated groups. These results suggest that *L. paracasei* VCare-360 can reverse the gut dysbiosis triggered by fungal vaginitis, restoring intestinal homeostasis through the regulation of core taxa like *Lactobacillus* and *Muribaculaceae* Zhu et al., 2024.

**Figure 11:**
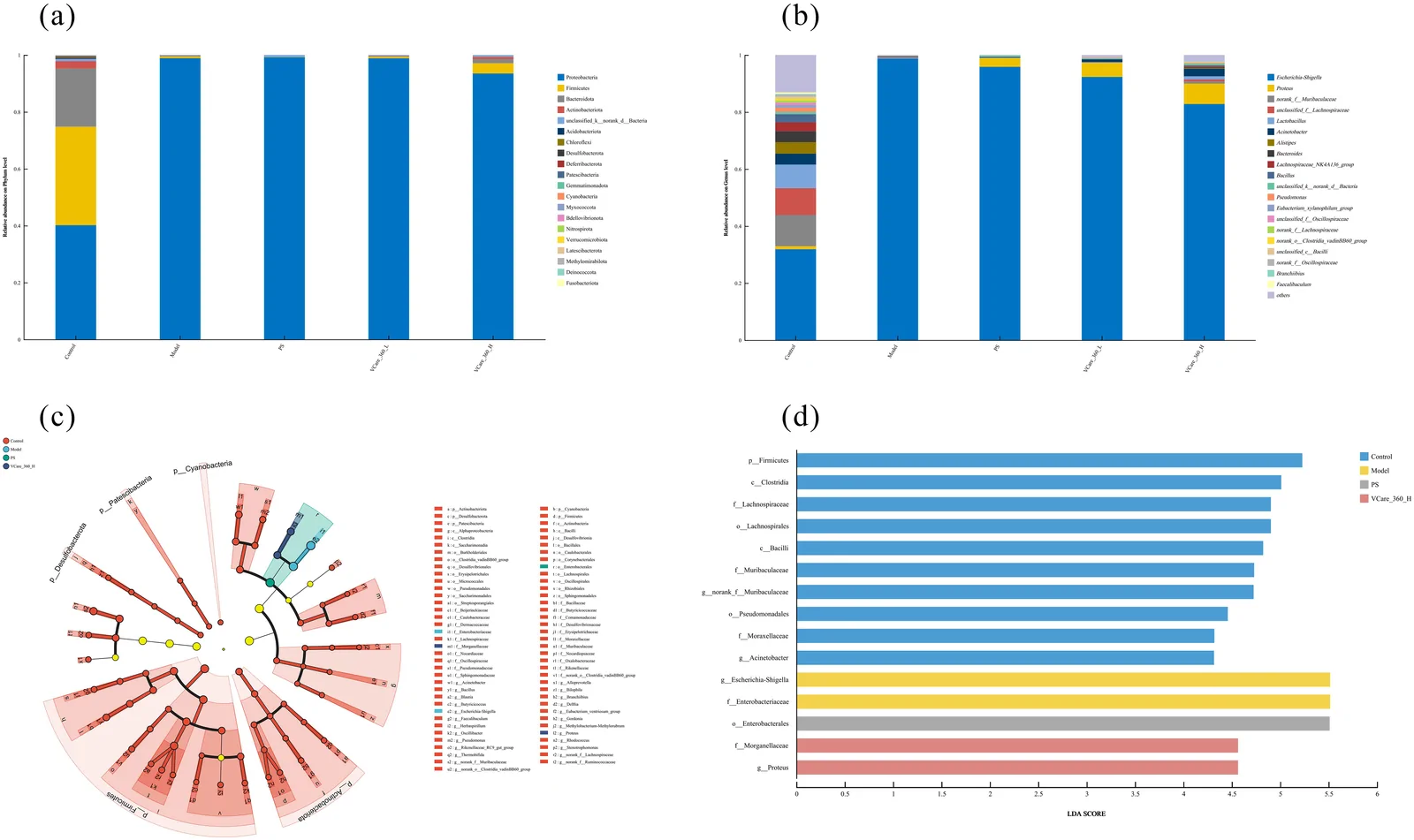
Analysis of vaginal microbiota composition and biomarkers. **(a)** Relative abundance of the vaginal microbiota at the phylum level in each group. **(b)** Relative abundance of the vaginal microbiota at the genus level in each group. **(c)** LEfSe cladogram representing the phylogenetic relationships of differentially abundant taxa among the Control, Model, PS, and VCare-360-H groups. The radius of the circle represents the relative abundance, and colors indicate the group with the highest abundance for that taxon. **(d)** LDA score distribution histogram identifying specific biomarker taxa with significant differences in abundance between groups. The length of the bar indicates the magnitude of the effect (LDA score), highlighting key bacterial taxa driving the differences between the experimental conditions.

**Figure 12:**
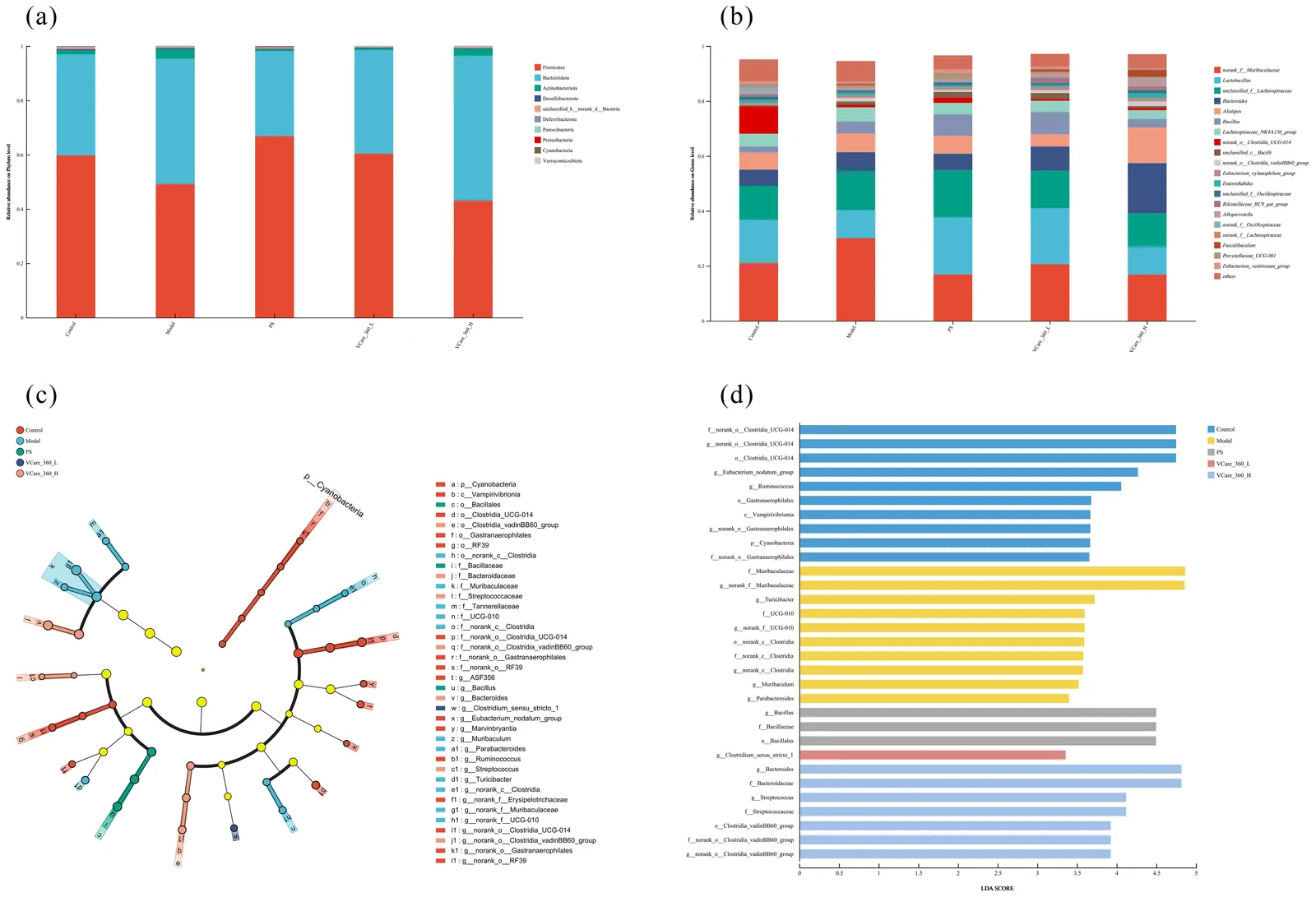
Analysis of fecal microbiota composition and biomarkers. **(a)** Relative abundance of the fecal microbiota at the phylum level in each group. **(b)** Relative abundance of the fecal microbiota at the genus level in each group. **(c)** LEfSe cladogram representing the phylogenetic relationships of differentially abundant taxa among the Control, Model, PS, VCare-360-L, and VCare-360-H groups. The radius of the circle represents the relative abundance, and colors indicate the group with the highest abundance for that taxon. **(d)** LDA score distribution histogram identifying specific biomarker taxa with significant differences in abundance between groups. The length of the bar indicates the magnitude of the effect (LDA score), highlighting key bacterial taxa driving the differences between the experimental conditions.

To assess the impact of vaginal inflammation and probiotic intervention on gut health, we analyzed the Gut Microbiome Health Index (GMHI) and Microbial Dysbiosis Index (MDI) (**Figure 13**). Consistent with the observed dysbiosis, the Model group exhibited significantly lower GMHI values compared to the Control group (*p* < 0.01, **Figure 13a**), indicating compromised gut microbial health. Conversely, the MDI was significantly elevated in the Model group relative to controls (*p* < 0.01, **Figure 13e**), further confirming the severity of dysbiosis induced by *C. albicans* infection. Intervention with *L. paracasei* VCare-360 effectively ameliorated these alterations. Both low and high doses of VCare-360 significantly increased GMHI scores compared to the Model group (*p* < 0.05, **Figures 13c, d**). Furthermore, the MDI was significantly reduced in both VCare-360 treatment groups (*p* < 0.01, **Figure 13e**), suggesting that VCare-360 supplementation helps restore gut microbial balance and alleviate dysbiosis associated with vaginal inflammation.

**Figure 13:**
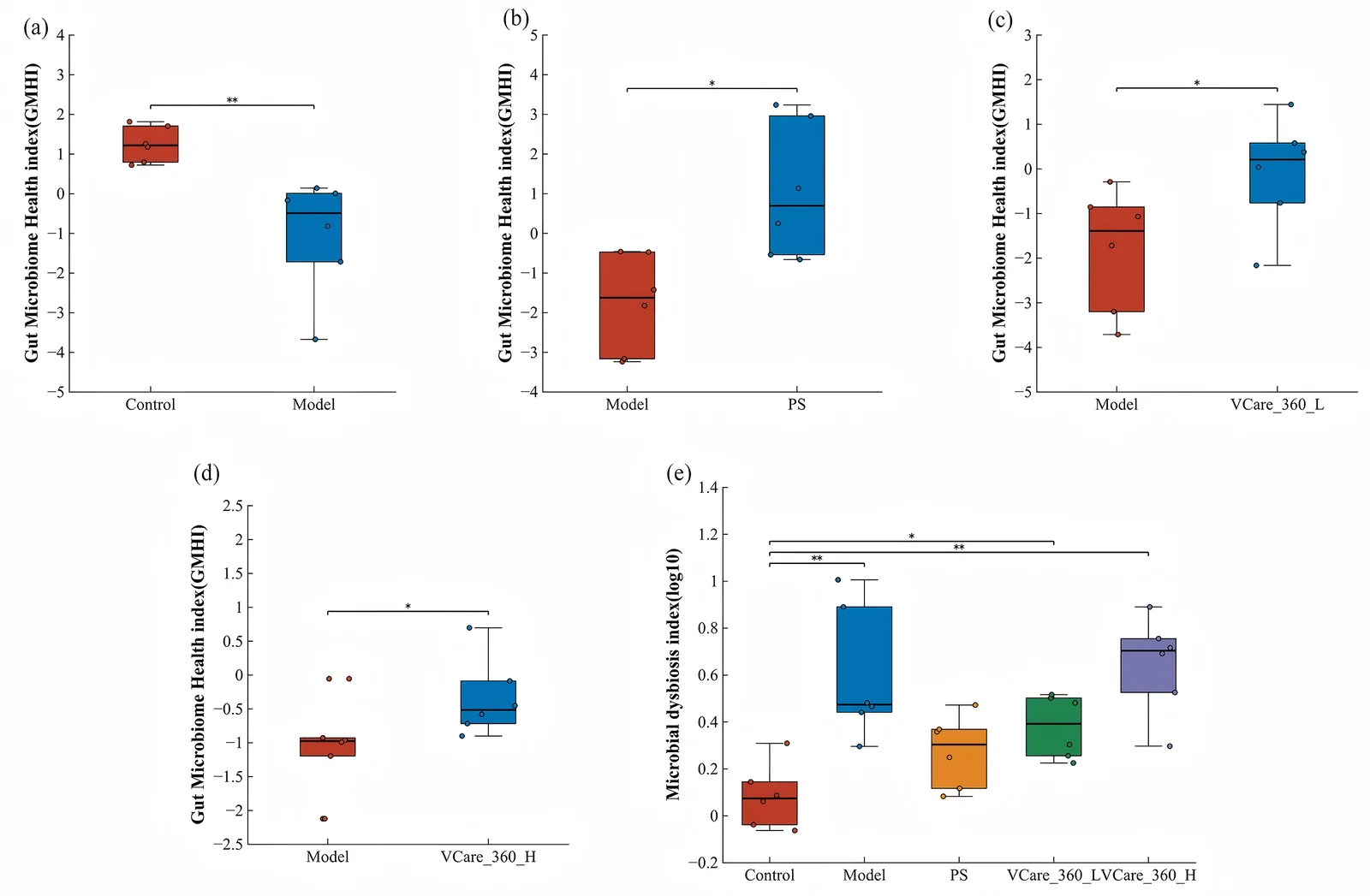
Assessment of gut microbial functional potential and dysbiosis. **(a)** Comparison of the Gut Microbiome Health Index (GMHI) between the Control and Model groups. **(b–d)** Pairwise comparisons of GMHI between the Model group and the treatment groups: **(b)** PS, **(c)** VCare-360-L, and **(d)** VCare-360-H. **(e)** Microbial Dysbiosis Index (MDI) across all experimental groups (Control, Model, PS, VCare-360-L, and VCare-360-H). Data are presented as box plots showing the median and interquartile range. Statistical significance is indicated by asterisks: *p ≤ 0.05, and **p ≤ 0.01.

## 4 Discussion

Maintaining vaginal microbial balance is key to preventing female reproductive illness, and probiotics are commonly applied to achieve this. This balance fosters a harmonious host-microbe interaction, creating a healthy vaginal ecosystem crucial for managing bacterial vaginosis, vulvovaginal candidiasis (VVC), trichomoniasis, and similar conditions (Han and Ren, 2021). Our findings collectively demonstrate that *L. paracasei* VCare-360 possesses a suite of characteristics conducive to mitigating VVC. *In vitro* assays demonstrated that this strain produces key antimicrobial metabolites, including hydrogen peroxide, lactic acid, and antimicrobial peptides, and exhibited a strong inhibitory effect against *C. albicans*, with an IC_50_ value of 0.65 (Andrade et al., 2022). These properties are fundamental to establishing a protective vaginal environment, as a healthy vaginal microbiota is typically characterized by low pH and the presence of H_2_O_2_, which collectively suppress the overgrowth of opportunistic pathogens such as *C. albicans*. It is the key to the self-cleaning ability of the vagina (Servin, 2004; Haya et al., 2014; Razzak et al., 2011). According to David Jose Delgado-Diaz et al., the lactic acid generated by cervicovaginal *Lactobacilli* plays a role in modulating genital epithelial barrier function, including increasing the expression of intercellular junctional molecules (Delgado-Diaz et al., 2022).Thus, the initial biochemical and antimicrobial profiles of *L. paracasei* VCare-360 align with the prerequisites for a successful vaginal probiotic intervention.

Beyond its direct antimicrobial effects, we examined how the probiotic interacts with vaginal epithelial cells, an interaction vital for successful colonization and the subsequent protective role it can play in the vaginal environment. The adherence of *L. paracasei* VCare-360 to the vaginal epithelium VK2/E6E7 facilitates the formation of a protective bacterial biofilm, which may play a key role in preventing pathogen colonization on the mucosal surface. In this study, *L. paracasei* VCare-360 markedly reduced *C. albicans* adhesion to the VK2/E6E7 cells, with adhesion indices of 41.75 ± 13.14 CFU/cell in the control group versus 10.95 ± 4.46 CFU/cell in the VCare-360 group. Furthermore, *Lactobacillus* were believed to support the maintenance of a healthy vaginal epithelial environment, thereby enhancing the resilience of the vaginal ecosystem against potential pathogens (Pi et al., 2011). Moreover, it demonstrated a strong capacity to displace pre-attached *C. albicans*, suggesting a potent competitive exclusion effect. Alessandra Pino’ s findings demonstrated that *Lactobacilli* strains effectively inhibited pathogenic bacteria through mechanisms of exclusion and displacement. Notably, *L. rhamnosus* PBE 07 and PBE 11 exhibited particularly high levels of inhibition, exceeding 50% against all tested pathogens (*C. albicans* ATCC 10231, *C. glabrata* ATCC 90030, *P. aeruginosa* DSM 3227, et al.) (Pino et al., 2024). Upon encountering *C. albicans*, vaginal epithelial cells initiate a defense response by secreting proinflammatory cytokines, thereby recruiting host defenses to the site of infection (McGowin et al., 2009). Antigens from *C. albicans* stimulate vaginal epithelial cell to produce inflammatory cytokines, specifically TNF-α, IL-6 and IL-8 (Schaller et al., 2006). In contrast, *L. paracasei* VCare-360 treatment led to a reduction in the expression levels of these inflammatory cytokines, indicating a mitigating effect on the inflammation caused by the fungal antigens.

Notably, the safety of oral supplementation with this strain has been formally validated. We have completed preliminary tests covering hemolytic activity detection, antibiotic susceptibility analysis, and *in vivo* pathogenicity assessment. No abnormal clinical signs, body weight loss, or mortality were observed during the 21-day observation period, which demonstrates its excellent biosafety profile. These results confirm that *L. paracasei* VCare-360 meets the safety criteria for probiotic strains (Han et al., 2025). Emerging research indicates that the gastrointestinal microbiota is interconnected with the vagina, establishing bidirectional communication pathways via the “vagina-gut axis” (Salazar et al., 2022; Takada et al., 2023). Upon oral administration, probiotic strains have the capacity to translocate to distinct colonization niches, such as the vagina, where they exert specific clinical effects (Reid et al., 2003; Morelli et al., 2004; Reid et al., 2001). Ang X. Y et al (Ang et al., 2023). reported that *Lactobacillus* play a protective role in maintaining a balanced vaginal and gut microbiota during vaginal candidiasis, thereby preventing dysbiosis and fostering a healthier microbial environment. In a randomized, double-blind, placebo-controlled trial, an eight-week course of probiotic *Lactobacilli* treatment was shown to be helpful for pregnant women, particularly in alleviating VVC symptoms and reducing the recurrence of VVC. Their findings suggest that probiotics may represent a promising therapeutic strategy for managing vaginal candidiasis through modulation of the gut-vaginal axis and enhancement of microbial resilience. In the context animal models, *Lactobacillus crispatus* CCFM1339 has been shown to exert notable anti-inflammatory effects, as indicated by a significant increase in IL-10 production, thereby ameliorating vaginal inflammation in mice. Immunological analysis further revealed that treatment with *L. crispatus* CCFM1339 elevated secretory immunoglobulin A (sIgA) levels in vaginal tissues (rising from 405.56 to 740.62 ng/mL) and concurrently reduced IL-17 gene expression. Critically, this probiotic also modulated the vaginal microbiota by increasing the abundance of beneficial lactic acid bacteria while decreasing the numbers of potentially pathogenic *Enterobacteriaceae* and *Enterococcus* (Huang et al., 2024). Studies have shown that oral administration of a five-strain *Lactobacillus* mixture (LM5) at a dose of 5×10^9^ CFU/mouse inhibited *G. vaginalis* proliferation in the vaginal tract, while also significantly reducing myeloperoxidase activity and levels of pro-inflammatory cytokine. Histopathological analysis further revealed that LM5 effectively suppressed the exfoliation of vaginal epithelial cells, indicating its potential to alleviate bacterial vaginosis-associated inflammation and tissue damage (Liu et al., 2023). In the present study, for the *L. paracasei* VCare-360 intervention group, the weight recovery was used as the primary indicator of efficacy, indicating that the systemic stress caused by the infection was alleciated, and the overall health condition of the mice improved. Furthermore, *L. paracasei* were detected in the vaginal samples of mice following oral administration. Moreover, the successful colonization of *L. paracasei* in the vagina following oral administration suggests that it may ameliorate vulvovaginal candidiasis through competitive adhesion. To corroborate this hypothesis, our *in vitro* cell assays clearly demonstrated that *L. paracasei* VCare-360 significantly reduced the adherence of *C. albicans* to vaginal epithelial cells. Collectively, these *in vivo* and *in vitro* finding indicate that *L. paracasei* VCare-360 significantly suppressed *C. albicans* colonization in the vagina, with the strongest effect seen in the concentration of 10^9^ CFU/mL. This protective effect may be primarily mediated by the production of antifungal metabolites such as hydrogen peroxide, lactic acid and bacteriocins, which are known to inhibit fungal growth and contribute to the maintenance of a healthy vaginal microenvironment, as was previously reported for other probiotics (Han and Ren, 2021). We demonstrated that administration of *L. paracasei* VCare-360 at a concentration of 10^9^ CFU/mL in BALB/c mice with vulvovaginal candidiasis inhibited inflammation by modulating the levels of anti-inflammatory (IL-10, IL-4) cytokine and pro-inflammatory cytokine (IL-1β, IL-6, TNF-α). This finding is consistent with the results obtained by R Doug Wagner, who reported *L. rhamnosus* GR-1 and *L. reuteri* RC-14 suppressed expression of TNFα and IL-8 mRNA in *C. albicans* infected VK2/E6E7 cells (Wagner and Johnson, 2012). Neutrophils (polymorphonuclear leukocytes, PMNs) are key mediators in the pathogenesis of vaginal inflammation. Upon infection, S100 proteins stimulate the massive recruitment of PMNs into the vaginal lumen, triggering a robust inflammatory response (Sun et al., 2020). Myeloperoxidase (MPO), predominantly expressed in neutrophils, serves as a marker of neutrophil activation and inflammatory intensity. Our results suggest that *L. paracasei* VCare-360 might reduce the *C. albicans* burden, thereby reducing the expression of MPO, and ultimately alleviating the harmful inflammatory response. The vaginal microbiota, as a key component of the vaginal barrier, plays a critical role in maintaining vaginal health. Disruption of this microbial community can increase susceptibility to genitourinary infections and adverse pregnancy outcomes (France et al., 2022). In this study, after infected with *C. albicans*, the vaginal microecological balance was disrupted, with a dramatic increase in *Escherichia*-*Shigella* abundance, which accounts for more than 98% of the total microbial community. Additionally, infection with *C. albicans* decreased the relative abundance of Firmicutes and Bacteroidota. Following the intervention with *L. paracasei* VCare-360, an increased relative abundance of Firmicutes and Bacteroidota was observed, along with a higher proportion of the *Lactobacillus* genus. These alterations may be linked to early modifications in the intestinal mechanical and immune barriers. *L. crispatus* CCFM1339 can boost vaginal *Lactobacilli* abundance and reduce *Enterobacterium* and *Enterococcus*, showing its probiotic potential for vaginal barrier regulation (Huang et al., 2024). Similarly, the fecal microbiota also yielded consistent results. Following probiotic intervention with variable dosages, the abundance of the *Lactobacillus* genus was increased to varying degrees. Consequently, orally administered probiotic strains can translocate to extraintestinal sites, such as the vagina, where they may exert beneficial effects by modulating the local microbial composition and alleviating dysbiosis. This translocation may occur via ascending physical pathways, hematogenous dissemination, or lymphatic transport. These findings support the existence of a gut-vagina microbial axis, wherein intestinal microbiota modulation can influence vaginal health.

Despite these promising findings, several limitations of this study should be acknowledged. First, although we utilized a human vaginal epithelial cell line (VK2/E6E7) and a mice model to evaluate the efficacy of *L. paracasei* VCare-360, the complex physiological environment and microbiome interactions in humans differ from those in animal models. Therefore, the direct translatability of these results to clinical settings requires further verification. Future studies should focus on randomized controlled clinical trials to comprehensively evaluate the optimal dosage, and therapeutic efficacy of *L. paracasei* VCare-360 in women with VVC.

## 5 Conclusions

The findings revealed that *L. paracasei* VCare-360 exhibits therapeutic potential for vaginal candidiasis. Specifically, this strain significantly inhibits *C. albicans in vitro*, suppresses *C. albicans* adhesion and downregulates pro-inflammatory cytokines in cellular inflammation models, and successfully colonizes the vaginal tissues of mice-concurrently reducing pro-inflammatory cytokine levels in vaginal tissues and blood, and modulating vaginal and fecal microbiomes to restore health. Collectively, these results support the potential of oral administration of *L. paracasei* VCare-360 for ameliorating VVC.

## Data Availability

The original contributions presented in the study are included in the article/supplementary material, further inquiries can be directed to the corresponding authors.
